# A Premium of 100,000 Dollars

**Published:** 1883-11

**Authors:** 


### A PREMIUM OF $100,000 DOLLARS.

Dr. Wm. H. Doughty offered the following preamble and resolution before the American Health Asociation, Savannah, Ga.:

"WHEREAS, The insecurity to the public health from epidemic prevalence of yellow fever, is chiefly due to a total ignorance of the real cause of the disease, together with the circumstances and laws governing its generation, reproduction and spread; and,

"WHEREAS, The probability of said cause conveys the idea of physical qualities, perhaps, tangibility and possible ultimate identity by some means within reach of science; therefore, with a view to stimulate inquiry in this direction,

*Be it Resolved,* That the Congress of the U. S. be memorialized through the Secretary of this Association to offer a premium of $100,000, open to the world, for the discovery of the true cause or germ of yellow fever, or any certain means of effecting its prevention, destruction or harmless modification, the same to be subjected to such practical tests, under the direction of Congress, as will determine its real merits and efficiency."

This is a move, to say the least of it, of such substantial backing as savors of success. Such a discovery would be worth to every civilized country unspeakably more than the specified premium. But will Congress respond? If it contains much of such material as constitutes an average Georgia Legislature, it will not, if it knows itself. Nil desperandum, brother Doughty.

